# PROJECT RESPECT: EXPERIENCES OF SERIOUSLY ILL LGBTQ+ PATIENTS AND PARTNERS WITH THEIR HEALTH CARE PROVIDERS

**DOI:** 10.1093/geroni/igae098.0797

**Published:** 2024-12-31

**Authors:** Gary Stein, Cathy Berkman

**Affiliations:** Wurzweiler School of Social Work-Yeshiva University, New York, New York, United States; Fordham University, New York, New York, United States

## Abstract

Discrimination against LGBTQ+ persons in healthcare creates barriers to serious illness care, including patients avoiding or delaying necessary care, providers disrespecting wishes of surrogates, and adverse outcomes for patients and families. A cross-sectional mixed methods study using an online survey was used to determine the extent to which LGBTQ+ patients and spouses, partners, and widows experienced disrespectful or inadequate care due to sexual orientation or gender identity. A total of 290 LGBTQ+ patients and partners reported high levels of disrespectful and inadequate care. Patients reported these discriminatory actions from healthcare providers: insensitive to me as an LGBTQ+ person (35.2%); not aware of LGBTQ+ health needs (30%); made me feel judged for being LGBTQ+ (23.1%); were rude to me (20.7%); didn’t use my correct pronouns (20.3%); and disregarded my treatment decisions (19.7%). Partners reported they were denied access to their loved one in intensive care or emergency room (12.4%); were treated badly (12.1%); and that their decisions were not followed (10%). Black and Hispanic patients were two to four times more likely than non-Hispanic white patients to report discrimination. This study demonstrated high levels of disrespectful and inadequate care towards patients and partners due to being LGBTQ+, especially problematic for Black and Hispanic patients and those living in politically conservative regions. Recommendations include federal and state civil rights laws to prohibit LGBTQ+ discrimination, and institutional practices to address discrimination, including cultural sensitivity training for staff.

